# The Utility of Administrative Data in Understanding the COVID-19 Pandemic’s Impact on Child Maltreatment: Learning From the Scotland Experience

**DOI:** 10.1177/10775595221108661

**Published:** 2022-06-14

**Authors:** Alexander McTier, Joanna Soraghan

**Affiliations:** Evidence and Evaluation Specialist, 3527 University of Strathclyde, UK

**Keywords:** child maltreatment, child protection, COVID-19 pandemic, statistics, data

## Abstract

The COVID-19 pandemic and associated public health ‘stay at home’ restrictions have intensified familial risk factors. Children would appear to be at increased risk of harm and abuse, yet administrative data from the early months of the pandemic showed falling cases of child maltreatment. Using weekly administrative data from Scotland, UK that span the first 17 months of the pandemic, this article found that child maltreatment activity levels fluctuated as ‘stay at home’ restrictions changed. During lockdown periods, the number of children subject to Inter-agency Referral Discussion fell but a higher number of children were placed on the Child Protection Register. When restrictions were eased, the number of Inter-agency Referral Discussions increased but the number of children placed on the Child Protection Register fell. To explain the fluctuations, the article asserts that the pandemic’s impact on services’ ability to engage directly with children and families has been critical, but the limitations of administrative data in providing an accurate measure of child maltreatment levels also need to be recognised. The article advocates that analysis of administrative data is best done in tandem with wider quantitative and qualitative sources in order to understand the impact of crisis events on children and families.

## Introduction

The majority of abuse to children is carried out by family members in the homes that children live in (Brandon et al., 2020). The onset of the COVID-19 pandemic consequently led to concerns that children would be at risk of maltreatment. By adhering to ‘stay at home’ restrictions, and notwithstanding that some families valued the increased time together away from the pressures of school, work and commuting, the consensus was that familial risk factors would be exacerbated and intensified (Driscoll et al., 2021; Green, 2020; Rodriguez et al., 2021; Romanou & Belton, 2020; Sari et al., 2021). Worsening parental physical and mental health, increased instances of domestic violence, cramped household living arrangements, pressures and challenges of home schooling, increased unemployment, economic stress, hardship and food insecurity, increased social isolation, and limited access to family and wider supports were all highlighted (Brown et al., 2021; Cappa & Jijon, 2021; Connell & Strambler, 2021; Galea et al., 2020; Griffith, 2020; Hansen, 2020; Herrenkohl et al., 2021; Katz et al., 2021; Rapp et al., 2021; Rodriguez et al., 2021). It was feared that the impact of these risk factors *“may impede parenting abilities and increase the risk of using ineffective parenting strategies”* (Sari et al., 2021 pp1). Indeed, both parental self-reported and child helpline data point to an increase in verbal aggression, physical punishment such as spanking or hitting, and neglectful behaviours toward their children (He et al., 2021; Marmor et al., 2021; Rapp et al., 2021). Children spending more time online was also identified as a risk factor, with concerns that instances of online abuse (cyberbullying, inappropriate content, hate speech, harassment, and unwanted contact) would increase (Cappa & Jijon, 2021; Katz et al., 2021).

The multiple risk factors, allied to the experience of previous health emergencies and natural disasters (Brown et al., 2021; Hansen, 2020; Rapp et al., 2021; Rodriguez et al., 2021; Whaling et al., 2021), all pointed to fears that more children would experience neglect or physical, emotional and sexual abuse. The National Guidance for Child Protection in Scotland 2021 defines maltreatment as follows, *“Abuse and neglect are forms of maltreatment (…*and*…) may involve inflicting harm or failing to act to prevent harm. Children may be maltreated at home; within a family or peer network; in care placements; institutions or community settings; and in the online and digital environment”* (Scottish Government, 2021 pp12). Anxiety among children’s professionals about the children they were responsible for increased, not only for the children known to services as vulnerable but also for those in newly vulnerable, hidden groups (Marmor et al., 2021; Whaling et al., 2021). Such hidden groups included children experiencing exacerbated parental stress and mental ill-health, new exposure to domestic violence or higher levels of parental substance misuse (Driscoll et al., 2021; Sari et al., 2021).

However, counterintuitively, the available administrative child maltreatment data for the early months of the pandemic in 2020 suggested a different trajectory. Rather than increases, available data indicated that reports of child maltreatment fell during ‘lockdown’ periods (Herrenkohl et al., 2021; Marmor et al., 2021). Hansen’s (2020) analysis of monthly child maltreatment data in five US states found that data for the March-June period were lowest in 2020 compared to each of the four previous years. Brown et al. (2021) similarly found that several US states had seen an 18%–50% decrease in referrals to child protection systems for alleged maltreatment in 2020 compared to 2019. Whaling et al. (2021) found that case openings of families accessing child welfare preventive services in New York City declined by 49% from pre-COVID to COVID periods. In the UK, data for England showed that the number of referrals to children’s social work services was 17% lower for April to June 2020 than in April to June 2019 (Department for Education, 2021).

It is in this context that this article considers a new weekly administrative dataset in Scotland, UK – the Vulnerable Children and Young People Dataset – to critically discuss the value of administrative data in understanding the impact of the COVID-19 pandemic on child maltreatment levels. Using the dataset as a case example, the article contributes to the literature in two areas. First, using the weekly data from April 2020 to August 2021, the article presents longitudinal data across different phases of the pandemic, so moving beyond the existing international evidence that centres on the early months of the pandemic. The article’s second contribution is to consider the strengths and limitations of child maltreatment administrative data and consequently how the data can help and hinder service planning. These two contributions provide the structure to this article, before it concludes with a discussion of how administrative statistical data should be considered alongside other data sources to enable more comprehensive understanding of child maltreatment levels.

### Interpreting official administrative data on child maltreatment

The counterintuitive statistical trends referred to previously have led to an apparent re-awakening in the literature of the limitations of administrative child maltreatment data. Some papers have sought to explain the mismatch between the number of children recorded as experiencing child maltreatment and the anticipated heightened risks that children were likely to be experiencing. The closure of schools is, for example, referred to as a key factor. Teachers and wider school staff play a vital role in noticing and reporting instances of child maltreatment; indeed, child educators are mandated reporters of child maltreatment in some countries (Marmor et al., 2021; Rapp et al., 2021; Whaling et al., 2021). In Scotland, all professionals across education, health, social work, police and the third sector, as well as members of the public, are expected (though not mandated) to share with police or social work any concerns about possible harm to a child from abuse, neglect or exploitation (Scottish Government, 2021). School closures limit education staff’s opportunities to observe their pupils and consequently will have an impact on the number of child maltreatment incidents recorded in administrative data (Cappa & Jijon, 2021; Connell & Strambler, 2021; Herrenkohl et al., 2021; Katz et al., 2021; Rapp et al., 2021). This aligns with the long-established pattern of child maltreatment reports falling during school holiday periods when schools are closed, and the COVID-19 pandemic-enforced restrictions meant extended periods of school closures (Brown et al., 2021; Petrowski et al., 2021; Rodriguez et al., 2021; Whaling et al., 2021). The reduced ability of health visiting, routine paediatric medical care and family support services to observe for child maltreatment is another explanatory factor. Some staff were redeployed to frontline health roles and many health services switched to remote delivery, thus limiting the face-to-face visits and contact that is so crucial to noticing concerns and for disclosures to be made (Driscoll et al., 2021; Rapp et al., 2021).

In other papers, the counterintuitive statistical trends have highlighted the technical limitations of administrative child maltreatment data, particularly when collated at the national level. Many of these issues are longstanding and include the lack of real-time (monthly or weekly) data, the inconsistencies in indicators (and their definitions) collected across and within countries, gaps in the data (e.g. particularly relating to online abuse), the challenge of aggregate data hiding small but acute concentrations of maltreatment (e.g. in neighbourhoods affected by poverty or low quality housing), and that administrative data are only counts of child maltreatment incidents notified to and recorded by professionals (Barboza et al., 2020; Cappa & Jijon, 2021; Fluke et al. 2020; Hansen, 2020; Katz et al., 2021; Petrowski et al., 2021). The last point is fundamental as it means that administrative data provide only a partial picture of the full prevalence and experience of child maltreatment because the data only capture those incidents known to services (Marmor et al., 2021).

To overcome these limitations, the inclusion and consideration of wider intelligence sources beyond social work administrative data are encouraged as collectively these offer truer indications of the prevalence of child maltreatment (Fluke et al., 2020). From health, there has been evidence of increased attendances at emergency paediatric health services and increased instances of physical injuries to children since the onset of the COVID-19 pandemic (Katz et al., 2021; Loiseau et al., 2021; Rapp et al., 2021; Rodriguez et al., 2021). From third sector organisations, the analysis of child helpline data can be particularly instructive as they *‘serve as critical windows into the home, helping to make child distress and maltreatment more “visible” during a time when educational personnel, daycare providers, and physicians have limited contact with at-risk children’* (He et al., 2021 pp2). Research from France, UK and USA find that calls to child and family helplines increased significantly (Katz et al., 2021; Loiseau et al., 2021; Petrowski et al., 2021; Romanou & Belton, 2020).

Notwithstanding the limitations discussed above, Fluke et al. (2020) find there is often a reliance placed on administrative (social work) statistical data. This then presents a dilemma. Practitioners and academics alike are of the opinion that the pandemic and ‘stay at home’ restrictions have placed children at increased risk, yet (the limitations of) the administrative data collected mean that the hidden harm is not visible (Sari et al., 2021). The risk is that policy makers have not seen the predicament facing vulnerable children, and thus have not viewed meeting their needs as a priority (Katz et al., 2021).

Efforts have, however, been made to better understand and respond to the impact of the COVID-19 pandemic on children. Katz et al. (2021 pp.13) note that *‘many jurisdictions were able to respond rapidly to the pandemic and to put into place new policies and operating procedures with unprecedented speed’*. With reference to Scotland, Katz et al.‘s observation holds true with a number of COVID-19 pandemic innovations initiated. These include the use of video technologies for child-practitioner meetings, direct financial payments to families, and increased trust and autonomy in practitioners (CELCIS, 2021). At a more strategic level, there has been the establishment of a national Children and Families Collective Leadership Group (consisting of senior leader representation from national and local children’s sector services) and the introduction of the new weekly Vulnerable Children and Young Person statistical dataset to monitor the impact of the pandemic on children and young people.

## Method

### Data and Population

Administrative data from the weekly Vulnerable Children and Young Person Dataset were used. The dataset was a new, national dataset, developed and agreed by the Scottish Government, Scotland’s 32 local authorities and other key stakeholders in March 2020 in response to the COVID-19 pandemic. Its first collection was in April 2020, with collection undertaken by each local authority and Scotland’s national police force (Police Scotland). The data are submitted each week to the Scottish Government, which compiles, analyses and reports on the data via a weekly report and an online, public access data dashboard (see Scottish Government Vulnerable Children and Adult Protection tableau public website: https://public.tableau.com/app/profile/sg.eas.learninganalysis/viz/VulnerableChildrenandAdultProtection/Introduction). The key audiences for the data are the aforementioned national Children and Families Collective Leadership Group, chief executive officers across Scotland’s local authorities, health boards and police divisions, and senior managers in local authority social work departments.

Data are publicly available for analysis from April 2020 to August 2021, thus providing a valuable and time-sensitive source of intelligence about the impact of the pandemic on Scotland’s vulnerable children and young people. Indeed, the 17-month period offered by the dataset is a key contribution to the academic literature, as the COVID-19 pandemic literature has to date largely centred on the initial months of the pandemic.

### Variables

Set out in Table 1, the weekly Vulnerable Children and Young Person Dataset consists of 13 indicators spanning children and young people subject to Scotland’s child protection processes or in alternative care arrangements. Many of the indicators previously existed at the local authority level but this was the first time the indicators were brought together as a weekly national dataset. This article focuses on the child protection indicators outlined in Table 1 to understand how the number of reported incidents of child maltreatment in Scotland has changed from April 2020 to August 2021. Specifically, the article analyses the weekly data for Scotland on the number of child protection concern reports generated by Police Scotland; the number of children subject to Inter-agency Referral Discussion; the number of children (including unborn babies) placed on the child protection register; the number of children who were de-registered from the child protection register; and the total number of children on the child protection register at the end of each week.

**Table 1.** Vulnerable Children and Young People Dataset Indicators.

Theme	Indicator	Data Provider
Child protection activity	Number of child wellbeing concern reports generated by police scotland	Police scotland
	Number of child protection concern reports generated by police scotland	
	Number of children subject to inter-agency referral discussion	
	Number of children (including unborn babies) placed on the child protection register in the last week	Local authorities
	Number of children placed on the child protection register in the last week for whom domestic abuse was a significant factor	
	Number of children who were de-registered from the child protection register in the last week	
	Number of child protection orders granted in the last week	
Looked after children	Number of children starting to be looked after at home in the last week	Local authorities
	Number of children starting to be looked after away from home in the last week	
Professional contact with children	Number of children on the child protection register: a) Seen face-to-face by a professional in the last 2 weeks	Local authorities
	b) In total in your local authority at the end of the week	
	Number of children with multi-agency plans: a) Contacted by a professional in the last week	
	b) In total in your local authority at the end of the week	
	Number of care experienced young people eligible for aftercare a) contacted by a professional in the last 2 weeks	
	b) In total in your local authority at the end of the week	
Missing children	Number of children reported missing to police by social work in the last week a) who are looked after at home	Police scotland
	b) Who are looked after away from home	
	c) Other children	

**Figure 1.**
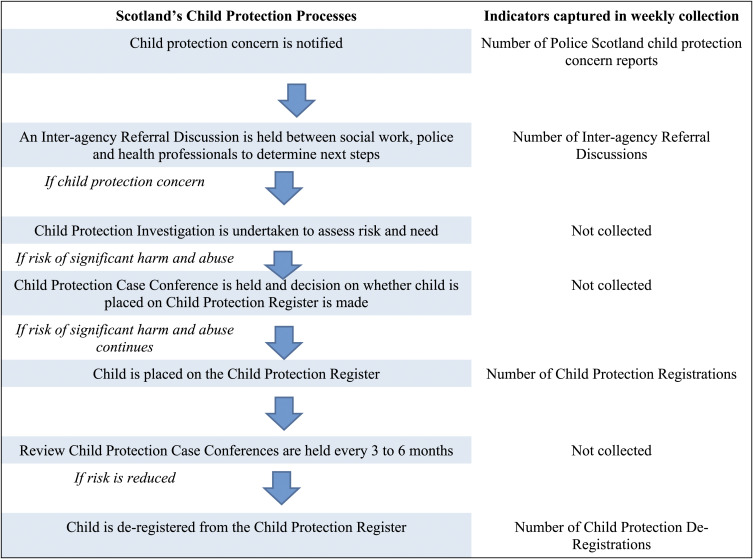
Child protection processes in scotland and alignment with weekly indicators collected.

To contextualise these indicators, Figure 1 provides an overview of how Scotland’s child protection processes are captured via the weekly indicators.

### Data Analysis

The data are first presented as time series charts, thereby deliberately mirroring how national and local children’s services leaders receive the data, and descriptive analysis is used to identify emergent trends and patterns. Second, the statistical significance of these emergent trends and patterns are tested for by the application of Wilcoxon Rank-Sum tests. This non-parametric alternative to the independent t-test was selected due to the divergence of the data from normality in several instances. Across both analytical approaches, key periods of the COVID-19 pandemic in Scotland have been referred to. The periods are: ‘first lockdown’ (April-June 2020), ‘phased re-opening’ (July to mid-December 2020), ‘second lockdown’ (mid-December 2020 to March 2021), and ‘opening under vaccination’ (April-August 2021). Data from the two lockdown and two re-opening periods were then grouped and compared when testing for statistically significant fluctuations in activity levels. During the two lockdown periods, schools were closed for most children but ‘community support hubs’ were open during these periods for vulnerable children and for children whose parents were frontline or key workers. In person contact and observation of some children during lockdown periods were therefore possible, but only 1% of all children attended these hubs (Couper-Kenney & Riddell, 2021) and the professionals staffing the hubs (such as early years practitioners or teachers) may not have previously worked with the children.

### Descriptive Results

**Figure 2.**
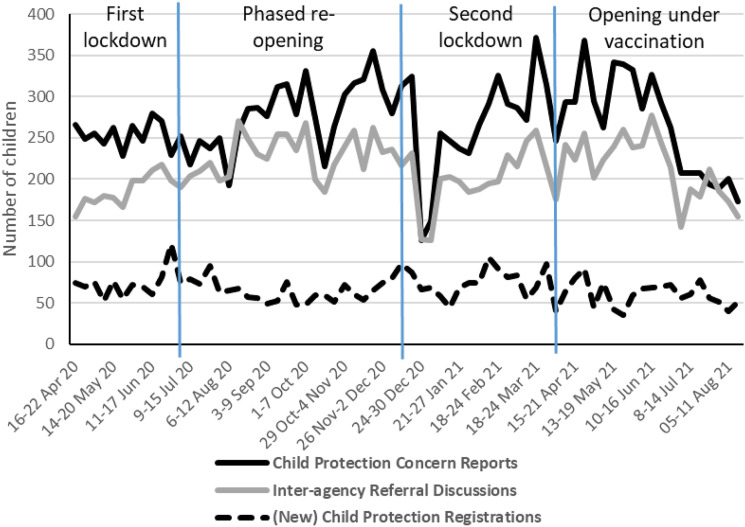
Number of weekly Child Protection Concern Reports, Inter-agency Referral Discussions and Child Protection Registrations; Scotland. *Source:* Scottish Government Vulnerable Children and Young People Dataset.

The analysis begins by considering three child maltreatment indicators: two relating to earlier child protection processes (number of Police Scotland child protection concern reports and number of Inter-agency Referral Discussions); the other (number of new child protection registrations) being an outcome of Inter-agency Referral Discussions, Investigations and Case Conferences, so a later stage indicator. They are presented in the same chart (see Figure 2) as a relationship should exist between them, i.e. an increase in earlier child protection processes would be expected to result in an increase in the number of child protection registrations.

Focusing first on the number of Police Scotland child protection concern reports and number of Inter-agency Referral Discussions, Figure 2 appears to show that numbers were comparatively low in the ‘first lockdown’, increased in ‘phased reopening’, before falling and stabilising in the ‘second lockdown’ and ‘opening under vaccination’ periods. At the same time, Figure 2 also points towards seasonal fluctuations and, specifically, fluctuations tied to school holiday periods that are long-established (Brown et al., 2021; Petrowski et al., 2021). There were upturns in activity when Scotland’s schools re-opened in mid-August 2020 and mid-April 2021; and dips at the start of July in 2020 and 2021 as Scotland’s summer school holidays start, mid-October 2020 for school half-term, mid-December 2020 as the Christmas school holidays start, and in April 2021 for the Easter school holidays. The upturn in activity at the end of lockdown periods could be interpreted as (lockdown-delayed) identifications of and responses to children’s needs and risks as they have become visible to education staff. However, attributing the increase in activity to the COVID-19 pandemic is made more difficult because Scotland’s lockdown periods coincided with school calendars.

### Children placed, on and leaving the Child Protection Register

**Figure 3.**
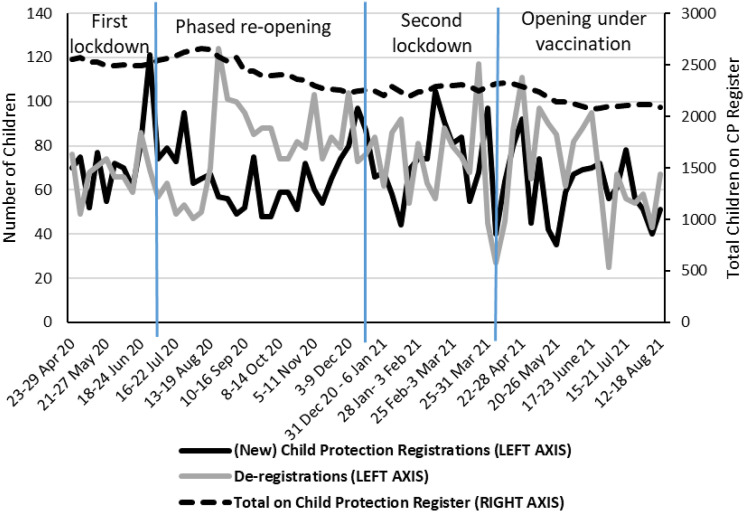
Number of (new) child protection registrations and de-registrations, and number of total children on the child protection register; scotland. *Source:* Scottish government vulnerable children and young people dataset.

Scotland’s Vulnerable Children and Young Person Dataset also provided data that helped to understand the dynamic nature of the child protection register. ‘In flow’ data were collected through the number of new child protection registers (presented in both Figure 2 and 3), ‘stock’ data in terms of the number of children on the child protection register at the end of the reporting week, and ‘out flow’ data in the number of children de-registered over the course of that week. Figure 3 presents the three indicators together because of the dynamic relationship between them.

Beginning with the number of new child protection registrations, there was a notable peak at the end of the ‘first lockdown’ in June 2020 in the lead up to the summer school holidays. The spike in registrations would appear to pre-empt increased risks and concerns during the summer holidays when vulnerable children’s attendance at ‘community support hubs’ and their contact with professionals would reduce. Again the spike demonstrates how it is difficult to attribute trends in the data to the impact of the COVID-19 pandemic alone because Scotland’s restrictions coincided with school calendars. The number of registrations then continued to exceed the number of de-registrations throughout the summer school holidays as the ability to assess children’s safety continued to be inhibited. The total number of children on the Child Protection Register consequently increased from 2550 children in April 2020 to 2654 children in mid-August 2020.

During ‘phased reopening’, two key patterns in the data emerge. First, and counter-intuitively, Figure 2 shows that the number of new child protection registrations reduced at a time when early stage child protection activity increased. Second, Figure 3 shows that the number of de-registrations were consistently higher than the number of registrations. The second pattern can be explained by the easing of COVID restrictions enabling professionals to more fully assess the safety and protection of children registered, and Review Child Protection Case Conferences (facilitated by more confident use of digital technologies) resumed to allow decisions on de-registrations. The number of children on the Child Protection register fell as a result. Since then, and notwithstanding weekly fluctuations, distinctive trends are less evident but overall the number of de-registrations has outstripped the number of registrations, meaning the total number of children on the Child Protection Register was 18% lower in August 2021 than at the onset of the pandemic in April 2020.

### Statistical Results

The descriptive analysis enabled by the time series charts appears to show some key and divergent trends in the data across the different periods of the COVID-19 pandemic in Scotland. However, it is also important to acknowledge that the charts are difficult to interpret with confidence because of the week-to-week fluctuations in the data and the lack of pre-pandemic, baseline data. In order to examine whether there were statistically significant differences in terms of child maltreatment activity between the combined lockdown periods and combined re-opening periods, Wilcoxon Rank-Sum tests were applied.

Table 2 validates some of the trends identified in the descriptive analysis. The weekly number of Inter-agency Referral Discussions was found to be significantly lower during lockdown periods (*Mdn* = 197.5) than re-opening periods (*Mdn* = 223.5); W = 303, *p* = 0.001. Conversely, the weekly number of child protection registrations was significantly higher in lockdown periods (*Mdn* = 73.0) than periods of re-opening (*Mdn* = 62.0); W = 794, *p* = 0.007. These divergent trends resulted in a difference in the conversion rate from Inter-agency Referral Discussion leading to child protection registration. The average weekly conversion rate across the combined lockdown periods was 38.9% (*SD* = 9.9), compared to 28.9% (*SD* = 7.2) across the combined re-opening periods.

**Table 2:** Median weekly number of Child Protection Indicators by Lockdown and Re-Opening Periods; with statistical comparisons.

	Lockdown Periods (Apr-Jun 2020 and Mid-Dec 2020 to Mar 2021)	Re-Opening Periods (Jul to Mid-Dec 2020 and Apr-Aug 2021)	Test-Statistic	*p*-value
N	26	44	--	--
Weekly child protection concern reports	264.0	279.5	W = 484.5	.290
Weekly inter-agency referral discussions	197.5	223.5	W = 303.0	.001
Weekly child protection registrations	73.0	62.0	W = 794.0	.007
Weekly child protection de-registrations	70.0	74.0	W = 520	.531

Items in bold where *p*<0.05.

*p*-values obtained from Wilcoxon Rank-Sum tests.

The number of Police Scotland child protection concern reports was slightly higher on average in re-opening periods (*Mdn* = 279.5) as opposed to lockown periods (*Mdn* = 264.0), although this was not found to be statistically significant (*p* = .29). Similarly, the difference in the number of child protection de-registrations in lockdown (*Mdn* = 70.0) compared to re-opening periods (*Mdn* = 74.0) was not found to be significant (*p* = .53). The relative stability in the level of de-registrations, in tandem with the decrease in child protection registrations during re-opening, explains the fall in the number of children on the Child Protection Register.

## Discussion

### Vulnerable children experiencing ‘hidden harm’?

The article used administrative data to examine child protection activity levels in Scotland during the first 17 months of the COVID-19 pandemic. Findings show that there have been differing trends that coincide with different periods of Scotland’s response to the COVID-19 pandemic. During lockdown periods, the number of children subject to Inter-agency Referral Discussion fell but a higher number of children were placed on the Child Protection Register. When restrictions were eased, the number of Inter-agency Referral Discussions increased but the number of children placed on the Child Protection Register fell.

Of these two patterns, the data for the lockdown periods more clearly align to the concept of hidden harm as they point to maltreatment not being identified and referred to child protection processes. Drawing on a parallel research study for Scotland’s Children and Families Collective Leadership Group (McTier & Sills, 2021), the views of senior social work managers representing seven multi-agency child protection committees that reflected the diversity of Scotland’s geography and population (Aberdeenshire, Argyll and Bute, Dumfries and Galloway, Dundee city, Glasgow city, North Ayrshire, and Na h-Eileanan Siar) attributed

Scotland’s experience of a reduction in the number of Inter-agency Referral Discussions to professionals having less contact with vulnerable children. Education, health and third sector family support services were all disrupted, so inhibiting professionals’ ability to work face-to-face with children and families and identify the maltreatment children were exposed to.

While the number of children subject to Inter-agency Referral Discussion was lower in lockdown periods, a higher number of children were placed on the Child Protection Register. This high conversion rate was interpreted by social work managers as a consequence of many of the children referred having more complex needs compared to pre-pandemic times (McTier & Sills, 2021). These views were reinforced by research conducted by third sector providers in Scotland that found the main concerns to be deteriorations of children’s mental health (in some cases manifesting to eating disorders, self-harm and suicidal thoughts), parental mental health, domestic abuse, problematic parental alcohol or substance use, neglect, and emotional abuse (Scottish Government, 2020; NSPCC, 2021). All were commonly identified concerns before the COVID-19 pandemic but these concerns had become more prevalent with family relationships straining under the heightened pandemic-related pressures related to poverty, job security and welfare payments (Independent Food Aid Network, 2020; NSPCC, 2021; Scottish Government, 2020). Social work managers also viewed the disruption of lockdown restrictions to preventative third sector family support services as a key contributory factor as services were less able to work with children and families before their needs escalate (McTier & Sills, 2021).

During periods of re-opening, a different pattern was evident in Scotland: one of increased Inter-agency Referral Discussions but fewer child protection registrations. The higher number of Inter-agency Referral Discussions can be explained by levels of contact with children increasing, so boosting visibility of maltreatment, but also due to professionals using Inter-agency Referral Discussions as a mechanism for gaining assurance from other professionals of children’s safety (McTier & Sills, 2021). Indeed, social work managers found that some professionals used Inter-agency Referral Discussions as an alternative to the informal, team-based case discussion with colleagues of pre-pandemic times (McTier & Sills, 2021). The increase in Inter-agency Referral Discussions did not, however, translate to increasing numbers of child protection registrations and instead a decrease in registrations was witnessed in re-opening periods. Social work managers explained this by finding that, on child protection investigation, the concerns were not found to place the child or young person at risk of significant harm or abuse (McTier & Sills, 2021). By not meeting the ‘risk of significant harm or abuse’ threshold, many cases did not progress to a Child Protection Case Conference and then child protection registration. Instead, children and families were directed to other types of targeted support via a single- or multi-agency Getting It Right For Every Child (GIRFEC) (i.e. a non-child protection) response.

In summary and in response to the question of ‘hidden harm’, there was assurance among Scotland’s children’s services that the needs of children and families already known to social work services were being met (McTier & Sills, 2021). The weekly professional contact data gathered as part of the weekly Vulnerable Children and Young Person Dataset and their scrutiny of local child protection processes and decision making were critical to this. However, in line with Driscoll et al. (2021), Marmor et al. (2021), Sari et al. (2021) and Whaling et al., 2021, there were concerns about ‘new families’ that had not previously been involved with social work services and may not actively reach out to them (McTier & Sills, 2021). These ‘new families’ were: new parents who had not had access to family supports and parent and toddler groups (Sari et al., 2021); families struggling financially but temporarily supported and ‘hidden’ by furlough employment schemes or local direct family payments initiatives (e.g. via local authorities and third sector organisations); and families – particularly those with a disability – that had been shielding and may struggle to re-engage with society as restrictions ease. A further point is that if these ‘new families’ did require support, it would take time and resource to fully assess and understand their strengths and needs as social work services had had no previous contact with them (McTier & Sills, 2021).

### Opportunities and challenges of administrative data

This article also helps us to critically consider the opportunities and challenges that administrative data offer in understanding levels of child maltreatment. Beginning with the opportunities, the Scotland experience shows how quickly a new, national statistical dataset can be established when the conditions are right. Referring again to Katz et al.‘s (2021 pp.13) assertion that *‘many jurisdictions were able to respond rapidly to the pandemic and to put into place new policies and operating procedures with unprecedented speed’*, the speed of the dataset’s development, agreement and then collection was striking. While acknowledging that many of the indicators did previously exist at the local authority level, the first collation of the weekly data by all 32 of Scotland’s local authorities and Police Scotland spanned just 4 weeks, so reflecting the urgency across the sector to respond to the COVID-19 pandemic. Executive level decisions were taken quickly and data officers, like their professional care giving colleagues, were committed to supporting children and families as effectively and responsively as they could.

A further point of learning from the Scotland dataset was that the pandemic made data available that were not previously used or reported on. For example, wider stakeholders did not know that Police Scotland held weekly data at a local authority area level on the number of child wellbeing concern reports, child protection concern reports and Inter-agency Referral Discussions – all of which addressed previous data gaps relating to earlier stage child protection activity. In a similar vein, contact data between professionals and vulnerable children and young people were not widely reported. These were welcomed by children’s services leaders as they provided some assurance over whether vulnerable children and young people were being contacted and their support needs discussed. The bringing together of new and existing local indicators demonstrated a creativity to the data collated nationally, resulting in a weekly dataset that helped national and local children’s services leaders to monitor the impact of the COVID-19 pandemic on child protection activity levels and raised questions for further investigation through quality assurance activity.

There are, however, also notes of caution based on the Scotland experience. First, the Vulnerable Children and Young People Dataset did not provide comprehensive data. As portrayed in Figure 1, data on the number of child protection investigations and case conferences were absent. These indicators would have helped to understand variations in the conversion rate between Inter-agency Referral Discussion and registration. More pertinent however was the absence of wider intelligence sources, not least child health and child helpline data, which would have contextualised the (predominantly social work) administrative child maltreatment data. A further observation is that the dataset did not offer the granular intelligence to identify small but acute concentrations of maltreatment (e.g. variations by age, gender, disability, locality, and concerns type) (Barboza et al., 2020). Whaling et al. (2021 pp7), for example, note the *‘accurate, timely, and geo-coded documentation of COVID-19 cases has been critical for steering the response to the virus. We argue that similar standards are required for child maltreatment service use during COVID-19’*.

Second, the administrative data collated within Scotland’s Vulnerable Children and Young People Dataset do not capture the prevalence of child maltreatment. Instead the data counted specific activities within the child protection system relating to the notification, investigation and subsequent management of child maltreatment concerns. The pandemic and related public health restrictions exacerbated this longstanding data shortcoming as child maltreatment became less visible to the child protection system and its practitioners. It is important that policy makers and service managers appreciate this shortcoming and view administrative data as proxies of child maltreatment levels, as opposed to accurate measures of child maltreatment.

Third, there were reservations aired about the accuracy of the data provided. The weekly cycle of collection and submission to Scottish Government meant there was little opportunity to validate the data (noting full validation may only take place once per annum in preparation for the annual Children’s Social Work Statistics return to Scottish Government). Data officers were dependent on the accuracy of the data inputted into management information systems by their colleagues. If data points were incorrectly filled out or missing, the weekly reporting cycle did not allow these to be verified or addressed. There were also issues raised about the definitional precision of some of the indicators contained in the dataset. For example, ‘multi-agency plans’ and ‘contact by a professional’ were not fully defined and this led to local variations in interpretation and collection. Such definitional issues are understandable as the speed of the dataset’s development and implementation did not allow for consultation and testing across stakeholders to ensure fully specified indicator definitions were agreed.

Fourth, there were challenges related to the time series of the dataset. On the one hand, the lack of baseline data at the national level before April 2020 made it difficult to identify with confidence any changing patterns of maltreatment experienced by children (Cappa & Jijon, 2021; Hood et al., 2016). On the other, presenting the dataset to children’s services leaders in the form of time series charts only, such as in Figures 2 and 3, brought challenges in how to interpret the week-to-week fluctuations in the data. The application of statistical techniques, as deployed in Table 2, should be made available to policy makers and service managers so that they can more accurately understand key trends and make decisions firmly based in the statistical evidence.

## Conclusion

Scotland’s response to understanding the impact of the COVID-19 pandemic on children is an example of Katz et al.’s (2021) finding that many jurisdictions were able to put in place new policies and procedures with unprecedented speed. The weekly Vulnerable Children and Young Person Dataset was quickly established and is likely to continue given the pandemic’s lifecycle and its anticipated long-term, delayed impact on children. The dataset’s value lies in providing weekly data that can help children’s services leaders to monitor and quickly respond to the impacts of the pandemic in terms of the number of children identified as experiencing maltreatment. Indeed, such real time data are a valuable addition that complements the existing annual national statistical collection. For policy and research interests, there would seemingly be real benefit from maintaining the data collection on a long-term basis, particularly if the real time data can later be validated via the collation of the national statistical collection and then supplemented by the use of statistical techniques to more rigorously analyse the data.

However, this article has also raised a number of limitations relating to the dataset that means administrative data alone are insufficient. To more fully understand the impact of the pandemic on child maltreatment, there is a need to consider social work administrative data alongside wider sources of intelligence (Fluke et al., 2020; Petrowski et al., 2021). A systems approach is therefore proposed whereby indicators, which tend to measure individual parts of the system, are combined and triangulated to give a picture of the system as a whole (Fluke et al., 2020; Hood et al., 2016). Multi-agency statistical data from health, education, police, third sector and wider family partners are key sources, but so too is capturing the views and experiences of local children’s services and, if appropriate and meaningful, the views and experiences of children and families themselves via surveys and analyses of helpline data.
